# Hypothesis Decay?: Blood Lead–Fluoridation Link Not Confirmed

**Published:** 2006-01

**Authors:** John Tibbetts

Numerous studies of various populations have shown that adding fluoride to drinking water prevents dental decay. However, a 1999 study in Massachusetts and a 2000 study in New York reported associations between the use of silicofluoride compounds in community water systems and elevated blood lead (PbB) concentrations in children. Now a team of researchers has tested the hypothesis generated by the Massachusetts and New York studies against findings from two other studies and found no cause for concern **[*EHP* 114:130–134]**.

As of 2000, the Centers for Disease Control and Prevention estimated that more than 162 million Americans were receiving fluoridated water. In the United States, several agents are used for fluoridation, including silicofluoride compounds (sodium silicofluoride and hydrofluosilicic acid) and sodium fluoride. Researchers with the Massachusetts and New York studies hypothesized that the silicofluoride compounds in tap water might enhance lead leaching from pipes and increase lead absorption from the water itself. Elevated PbB concentrations in children are associated with a host of cognitive, developmental, and behavioral impairments so serious that lead-based paint was banned in the United States in 1978 and lead water pipe solder was banned in the 1980s.

The current research group evaluated the relationship between water fluoridation method and PbB concentrations in children by conducting a large-scale statistical analysis of two other preexisting studies: the 1992 Fluoridation Census and the Third National Health and Nutrition Examination Survey (NHANES III). In analyzing data from NHANES III and the 1992 Fluoridation Census, the team improved on prior analyses by log-transforming raw PbB concentration and by including information on possible confounding factors missing from the Massachusetts and New York studies. These included poverty status, urbanicity, duration of residence, and year in which the dwelling was built.

The NHANES III sample was comprehensive, representing more than 52 million U.S. children. This survey also oversampled young children, older adults, non-Hispanic blacks, and Mexican Americans to ensure that population estimates for these groups would be statistically reliable.

The team found that, overall, the PbB concentrations of children who lived in counties receiving silicofluorides did not differ significantly from the PbB concentrations of children living in counties without fluoridated water. This was true even when researchers controlled for the year in which children’s homes were built. Given these findings, the team states there is no support for concerns that silicofluorides in community water systems cause higher PbB concentrations in children.

However, the investigators acknowledge that their analysis has limitations. For example, NHANES III did not measure the lead content of drinking water consumed by study participants. Also, the team did not control for factors such as density of older housing, and they were unable to control for the solubility of lead in pipes affected by different temperatures and water hardness.

Because of these limitations, the investigators cannot completely rule out a link between water fluoridation method and lead uptake in children, particularly in children living in older dwellings. They speculate that other studies, possibly those including chemical investigation and animal toxicology, could yield additional valuable data. They conclude that efforts to prevent dental decay via the use of fluoridated drinking water should continue unless a causal effect of specific fluoridation methods on PbB concentration is demonstrated by additional research.

## Figures and Tables

**Figure:**
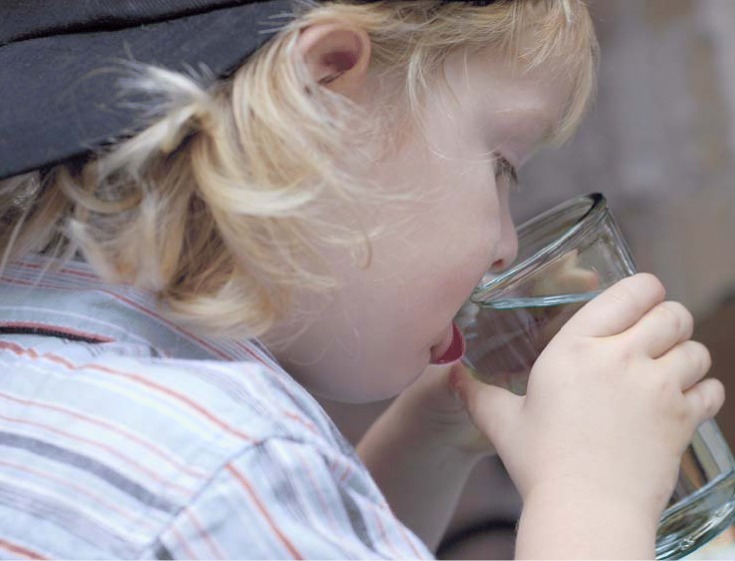
Refreshing news. Although some questions remain, a new data analysis fails to confirm fears that fluoridation of drinking water results in higher blood lead along with stronger teeth.

